# The Institutional Library

**Published:** 1920-06-05

**Authors:** 


### THE INSTITUTIONAL LIBRARY.

**Tuberculosis and Public Health.** By H. Hyslop Thomson, M.D., D.P.H. (Longmans, Green and Co. Pp. 104. 5s. net.)

This book, while advancing nothing very new in the way of theories and suggestions, presents the main facts of tuberculosis in its relation to the public health with clearness, cogency, and simplicity. Written primarily for medical men, Dr. Thomson's underlying effort to render it useful to the ordinary lay reader has been eminently successful. There is nothing not to be understood with ease by persons of average intelligence, and the book is of so practical a nature that it deserves a wide popularity amongst that great public without whose informed interest and co-operation the great battle never can be won.

Dr. Thomson insists very rightly that if the scourge is to be overcome attention must be focussed rather on prevention than on cure, and deals admirably with housing, milk, etc. In both these matters a vast amount remains to be done. In respect of housing, time must inevitably play an important part. But what of milk? A pure supply could be ensured inside of three months, and yet a mother cannot be reasonably sure she is not giving her child a lethal dose of tubercle bacilli without boiling and spoiling the food upon which the healthy development of the young so largely depends.

The treatment of the individual patient is dealt with in a comprehensive manner, the various schemes of dispensary, hospital, sanatorium, colony, etc., being discussed in their respective relations to one another.

We have only one quarrel with Dr. Thomson, which arises out of his statement (in the chapter on the necessity for early diagnosis), "In the diagnosis of pulmonary tuberculosis the general practitioner must still rely to a large extent on his powers of observation, his stethoscope, and the examination of the sputum." A more dangerous piece of advice it would be difficult to conceive. How often there is nothing to observe, and no sputum. This leaves the general practitioner with his stethoscope, by means of which he is to make a diagnosis of early phthisis—a condition whose auscultatory manifestations are so subtle, and indeed equivocal, that we are sure Dr. Thomson himself, though an expert, would be one of the last to give a patient a clean bill simply as the result of stethoscopic examination. The book merits a second edition, in which case we beg Dr. Thomson to amend his advice. Any patient suffering from persistent tiredness and feeling of slackness, together with a rectal temperature running a few points above normal after half an hour's rest in the evening, should be seen by a chest specialist, whether there be cough and sputum or not.

**Functional Nerve Disease.** Edited by H. Crichton Miller, M.D. (Oxford Medical Publications: Henry Frowde, and Hodder and Stoughton. 1920. Pp. 208. Price 8s. 6d. net.)

As is often the case with departments of medical science long neglected and then advanced by rapid strides through the labours of eager research workers, psychology is a subject whereof the general body of the medical profession has still somewhat hazy ideas. Many of the terms used by writers on psychological medicine carry no exact significance to the rest of us; nay, even fellow-specialists do not always attach the same shades of meaning to them. Much of what has been published is further obscured by the lack of the rare faculty of clear exposition on the part of authors endeavouring to explain their meaning; and some of these teachers are not unreasonably suspected of being by no means clear of what it is they want to expound. The war, however, has forced the subject into prominence; and there is a real want of an elementary textbook from which the intelligent practitioner or student shall understand the best of modern teachings as they are so far accepted by recognised authorities. In this monograph eleven authors have combined to epitomise war experience of psychoses or psychoneuroses so that the rest of the profession may understand. Broadly speaking, for there are individual divergences, the authors are of the Freudian school, though they avoid the excesses which have brought that system into a certain amount of disrepute. They advocate psycho-analysis, suggestion, hypnotism, and so on. Some of them are more helpful and intelligible than others; but, speaking for himself alone, the present reviewer confesses that they have not succeeded in dispelling the fog of confusion and ignorance of this subject which besets him, and that he does not feel any more competent than before to undertake the care of psychotic patients.

**The Link between the Practitioner and the Laboratory.** By Cavendish Fletcher, M.B., B.S., and Hugh McLean, B.A., B.C., D.P.H. (London: H. K. Lewis and Co., Ltd. 1920. Price 4s. 6d.)

As stated in the preface, the object of this little book is to enable practitioners to send to the pathologist "the right material in the right way." The text is written by two members of the staff of the Laboratories of Pathology and Public Health, an institution which is prepared to examine at fixed charges specimens sent to it from a distance. As a link between such central laboratories and the practising medical man the volume serves a reasonably useful purpose, but if the sender of specimens is even moderately *au fait* with the medical teachings of to-day he will hardly need such elementary advice as the bulk of the pages contain. If, too, he possess a mind which prefers to understand the reasons for the orders given to him, he will certainly have to consult a larger work on the subject.

**The Radiography of the Chest.** Vol I. Pulmonary Tuberculosis. By Walker Overend, M.A., M.D., B.Sc. Large octavo, pp. 119, with 99 radiographs and nine diagrams. (London: William Heinemann. N.D. 17s. 6d. net.)

This is a book of distinctly more than average merit. The inevitable progress of specialisation is seen in the devotion of a whole volume to the radiography of pulmonary tubercle; similarly abroad there are, we believe, radiologists for surgical tuberculosis alone. Now these technicians must be listened to very carefully and fully, not perhaps because of what they have done, as of what, granted even a slight improvement of their instrument, they may do. They have, indeed, had a great hand in the founding of our modern discovery of hilus tuberculosis, and very clearly they may ere long take a far bigger part in diagnosis at least. But at present the indications they go on seem indefinite. It is one of the few faults of the present work that we are not brought near to these indications. Certain shadows are

**The Institutional Library**—(*continued*).

pronounced to mean caseation, other opacities are dubbed infiltrations, others bronchiectasia. The not specially instructed eye makes out no clear differences between them, and one's heart is chilled with doubt. This may be partly because in a book one cannot see the original plates, and in this country the radiographic image, especially of lung conditions, loses at least 10 per cent. by reproduction in block form—Sauerbruck's or Jacobæus' illustrations seem as good as our originals. If the pulmonary radiologist would give us his criteria of differentiation—his physical signs, as it were—it would be helpful. The only other fault is that silicosis cases are almost absent, and these, owing to industrial reasons, are the ones most frequently $x$-rayed. But every tuberculosis specialist of the slightest ambition should work carefully through the ninety-nine radiograms here, with their descriptive text. He will rightly smile when activity or quiescence is diagnosed on the strength of the skiagraph, but he will realise afresh that he must keep a very watchful eye on the radiologist, who may soon rise on a flight far beyond our present level of knowledge.

**A Primer of Tropical Hygiene.** By Col. R. J. Blackham, C.B., C.M.G. (Bombay: G. Claridge and Co., Caxton House. 1919. Price One Rupee.)

The sixth edition of this little work is thoroughly revised and enlarged. It certainly deserves to be more widely known outside India, in that its teaching applies not only to that Empire but to our many colonies and dependencies situated in tropical or subtropical zones. At the present time, when the subject of continued overseas work is being considered by a large number of the younger medical generation, we can heartily recommend to them Colonel Blackham's little book as an excellent introduction to part at least of the studies which they must make.

**The Care of Sane Epileptic Children.** By J. Tylor Fox, M.A., M.D. (London: John Bale, Sons and Danielsson, Ltd. Pp. 59. 1s. 6d. net.)

In this little booklet Dr. Fox outlines the salient features of colony life. His impressions are the result of some eighteen months spent as Medical Superintendent of Lingfield, the colony founded in 1898 by the National Union for Christian Social Service. The study is confined to those patients under the age of sixteen, as after that the epileptic who has not made real progress tends to pass into the class of chronic case with established fit habit and gradually developing dementia.

The loss of family life entailed by prolonged residence in a colony is admitted to be a drawback when viewed superficially, but the disadvantages of home environment in the majority of cases are shown to be so serious and the advantages of institutional treatment so manifold that the objection loses all force.

The withdrawal of the child from the control of parents often nervously unstable and from an enforced brooding, indoor life, to an atmosphere of healthy open-air activity and expert supervision is of incalculable advantage to it. Of drugs Dr. Fox speaks doubtfully. The controversy regarding bromides remains unsettled. The administration of large doses was stopped at Lingfield years ago, and the results are certainly striking. In 1902 the population of the colony by years was 762, and the average of bromide per patient 10.8 ounces, with an average fit record of 98. By 1915, with a year population of 1,450, the bromide had been reduced to .45 ounce per patient, with an average fit record of 95!

As to outlook for these young patients, of 185 cases 58 were free from fits for the last two of the four years recorded.

Many other points of interest are dealt with, and all who have the charge of epileptic children would be well advised to read this booklet carefully.

**The British System of Physical Education.** By Beatrice E. Bear, M.B., A.P.T. With Foreword by Sir James Crichton Browne, M.D., F.R.S. (London: G. Bell and Sons, Ltd. Price 8s. 6d. net.)

The necessity of physical development in the education of the young never has been more vital than now, faced as we are with the very serious handicaps of post-war conditions. Miss Bear has rendered a signal service to the cause of physical education by setting forth as an ordered scheme the principles of the British system. The Swedish system, so largely in use still, has limitations and drawbacks as well as great merits. "Its progression is almost entirely in degrees of strength. The advanced work . . . whilst still remaining simple in construction, is extremely arduous." The outstanding quality of the British system is found in the progress of its exercises, rather in degrees of complexity than of strength, whilst it is equally beneficial from the point of view of health and physique. The increasing power of co-ordination attained means a poise, balance dexterity, and grace wholly admirable, whilst it also produces self-reliance, alertness, initiative, and courage—qualities whose merits cannot be overestimated. We have nothing but praise for the book, which is attractive in appearance and well arranged. The first three chapters describe the system, the classification of exercises, and the order of a lesson. The illustrations and diagrams of the succeeding parts are clear and well executed.

**Minor Surgery and Bandaging for the Use of House Surgeons, Dressers, and Junior Practitioners.** By Gwynne Williams, M.S., F.R.C.S., Assistant Surgeon, University College Hospital. (London: T. and A. Churchill, 7 Great Marlborough Street. 1920. Price 10s. 6d. net.)

This is a seventeenth edition, and as such lives up to the excellent reputation established by its predecessors. The whole has been thoroughly revised, to some extent rearranged, and the method of its incorporation of the lessons derived from the application of war surgery is to be thoroughly recommended.

**An Index of Symptoms with Diagnostic Methods.** By R. W. Leftwich, M.D. (London: John Murray, Albemarle Street, W. 1920.)

The seventh edition of this well-known work presents most notably many newly classified conditions arising out of the European War, and also a further list of unfamiliar diseases which have lately come under notice by the profession. Its good reputation is fully maintained.

**Half-past Twelve.** Dinner-hour Studies for the Odd Half-hours. By George W. Gough. (London: Sells, Ltd., 168 Fleet, E.C. 4. Price 1s.)

This pamphlet contains twenty-six short chapters on the elements of economics. It explains the nature of a cheque, the mechanism of exchange, investment, capital, rent, and the like in simple language which seeks not to lead the reader to any conclusion, for the last chapter has nothing to say, but to an understanding of the economic system in which he finds himself. As such it is useful, and should be welcome to those who do not know where to look for a simple guide to the elements of political economy.

